# Brain AT_1_ Receptor Involvement in Cognitive Decline and Neuroinflammation in Streptozotocin-Treated Rats

**DOI:** 10.3390/cimb48100998

**Published:** 2026-09-28

**Authors:** Mariela Fernanda Pérez, María de los Ángeles Marinzalda, Victoria Belén Occhieppo, Claudia Bregonzio, Gustavo Baiardi

**Affiliations:** 1 Instituto de Farmacología Experimental Córdoba (IFEC-CONICET), Departamento de Farmacología Otto Orsingher, Facultad de Ciencias Químicas, Universidad Nacional de Córdoba, Córdoba X5000, Argentina; 2 Laboratorio de Neurofarmacología, Instituto de Investigaciones Biológicas y Tecnológicas (IIBYT-CONICET), Córdoba X5000, Argentina

**Keywords:** brain angiotensin II, AT1 receptors, inflammation, oxidative stress, memory impairment, streptozotocin

## Abstract

Neuroinflammation and oxidative stress are involved in neuropathologies related to learning and memory decline. Angiotensin II contributes to neuroinflammation through angiotensin II type 1 receptors (AT1-R), leading to oxidative stress and release of pro-inflammatory mediators. This study aimed to evaluate the role of brain angiotensin II, via AT1-R, in streptozotocin-induced cognitive decline and neuroinflammation. For this purpose, male Wistar rats were treated with the AT1-R blocker candesartan cilexetil (3 mg/kg) for 25 days. On days 12 and 14, the animals received an intraventricular injection of streptozotocin (3 mg/kg) each day. On day 25, they were tested for spatial, working, and declarative memory through the Morris water maze, holeboard, and novel object recognition tests, respectively. The oxidative stress marker, malondialdehyde, was measured in isolated brain microvessels. Cerebrospinal fluid was collected for tumor necrosis factor-α determination. The volume of the lateral ventricles was evaluated for morphological changes. The AT1-R blocker pretreatment prevented streptozotocin-induced declines in declarative and working memory. No changes in spatial and long-term memory were observed. Streptozotocin increased malondialdehyde in isolated microvessels and tumor necrosis factor-α levels, which were prevented by candesartan administration. All these findings are consistent with the involvement of AT1-R in early streptozotocin-induced changes, with behavioral and inflammatory/morphological outcomes likely mediated by overlapping yet partially distinct mechanisms.

## 1. Introduction

Sporadic Alzheimer’s disease (sAD) is the most common form of dementia affecting elderly people worldwide. Its pathological mechanism remains unclear, but inflammation and oxidative stress appear to be the primary factors in the development and maintenance of the disease [1]. At the time of diagnosis, patients present with a plethora of common symptoms, with underlying processes already established in the brain, and sparse treatment options to prevent the characteristic memory impairment [1]. Intracerebroventricular (ICV) administration of streptozotocin (STZ) is a common experimental model used to simulate cognitive deficits observed in patients with sAD [2,3]. The STZ-injected animals undergo an insulin-resistant brain state associated with memory impairment and progressive cholinergic deficits, hypoglucose metabolism, and neurodegeneration that shares many features with sAD in humans [2,4,5]. Furthermore, there is evidence of increased oxidative stress markers in rodent brains following STZ [6,7]. In this sense, the antioxidant glutathione was decreased, and the lipid peroxidation marker, malondialdehyde (MDA), was found to be increased [8].

The extensive literature suggests that the dysregulation of the renin-angiotensin system acts as a primary driver in the pathogenesis of several brain disorders, including sAD. Clinical observations indicate that reducing systemic hypertension serves as a preventative measure against cognitive deterioration; however, it is still unclear whether particular categories of antihypertensive medications provide more effective neuroprotection (reviewed in [9]). Previous reports from our laboratory showed that brain Angiotensin II (Ang II), mainly through Ang II Type 1 receptors (AT1-R), plays a critical role in oxidative stress, neuroinflammation, and cognitive alterations in psychostimulant-induced animal models of schizophrenia. We observed that these alterations induced by ketamine or amphetamine exposure were prevented by candesartan cilexetil administration, an AT1-R antagonist, which is a widely used antihypertensive drug [10,11]. Regarding Ang II and sAD, both animal studies and clinical trials have yielded inconsistent results, often due to short follow-up times or low dementia incidence within the study groups. Coincidentally, long-term memory deficits induced by STZ administration in mice were attenuated by the AT1-R blocker telmisartan and by lisinopril, an angiotensin-converting enzyme inhibitor [6]. On the other hand, Tota et al. found that AT1-R plays a facilitative role in the memory deficit induced by ICV-administered STZ [12]. Discrepancies across reported findings likely emerge from variations in pharmacological administration windows, animal models employed, and specific methodological frameworks.

Considering all this evidence, the present research aims to characterize the role of AT1-R in cognitive responses and inflammatory processes induced by ICV-administered STZ, a rat model of sAD.

## 2. Materials and Methods

### 2.1. Animals

The study was performed in adult male Wistar rats, weighing 290–340 g. A total of 144 animals were used from the IFEC-CONICET, Department of Pharmacology, Faculty of Chemical Sciences’ vivarium. Animals were randomly distributed into groups, housed in plastic cages, and maintained under standard housing conditions (room temperature 21 °C ± 1 °C), with a 12 h light/dark cycle and free access to food and water. After surgery, the animals were housed in individual cages. Procedures involving animals were approved by the Animal Care and Use Committee of the Faculty of Chemical Sciences, National University of Córdoba, Argentina, Res. 270/18 (Approval date 7 March 2018), and renewed by Res. 2138/2022 (Approval date 18 October 2022) and Res. 1843/24 (Approval date 14 October 2024), in accordance with the NIH Guide for the Care and Use of Laboratory Animals. To minimize the number of animals used, those employed for the behavioral testing were randomly assigned to the biochemical analyses. The surgical procedure and the final disposition were performed under anesthesia. In all cases, the recommendations of the Animal Care and Use Local Committee were followed to minimize animal suffering.

### 2.2. Experimental Design

The experimental groups contained no more than two animals per litter and breeder to ensure a wide genetic background, and were treated according to the experimental protocol shown in Figure 1.

#### 2.2.1. Administration of Candesartan

To elucidate the AT1-R involvement in the STZ-induced alterations, the animals received the AT1-R blocker (AT1RB) or 0.1 N NaHCO_3_ as vehicle by gavage, once daily for 25 days. Based on our previous work [10,11,13], we used candesartan cilexetil (Phoenix Laboratories, Buenos Aires, Argentina), at a dose of 3 mg/kg. This is a widely used AT1-R antagonist that can cross the blood–brain barrier [14].

#### 2.2.2. Intracerebroventricular Administration of STZ

On day 8, the animals underwent stereotaxic surgery. The rats were anesthetized with ketamine-xylazine (75–5 mg/kg i.p.) and were fixed on the stereotaxic frame (Model 963 of 100 µm precision, David Kopf Instruments, Tujunga, CA, USA), and the skull was exposed. Steel cannulas (23G) were implanted into the lateral ventricles according to the following coordinates relative to bregma: anteroposterior −0.92 mm; lateral ± 1.5 mm; and dorsoventral 3.5 mm [15]. After 4 days of recovery, animals received two injections of STZ (3 mg/kg) dissolved in artificial cerebrospinal fluid (aCSF, 140 mM NaCl; 3 mM KCl; 1.2 mM CaCl_2_; 1 mM MgCl_2_; 1 mM Na_2_HPO_4_; 0.3 mM NaH_2_PO_4_; 3 mM D-glucose; pH 7.3) [3]. Administration was performed bilaterally, with an interval of 48 h (on days 12 and 14; [2]), using a stainless steel injection cannula (30G; 40 × 10.5 mm long) connected to a polyethylene tube (P50) mounted on a 25 µL Hamilton syringe (LOT#437742, Hamilton Company, Bellefonte, PA, USA) connected to a microinfusion pump, which allowed for the precise administration of the drug at a volume of 2 µL into each ventricle over a period of 2 min. When the cannulae are correctly positioned in the ventricles, a small amount of cerebrospinal fluid (CSF) will flow out. Only animals with cannulae in the proper position and free from occlusion were included in the analysis.

#### 2.2.3. Experimental Groups

Group 1: vehicle-artificial cerebrospinal fluid (Veh-aCSF).

Group 2: vehicle-streptozotocin (Veh-STZ).

Group 3: candesartan-artificial cerebrospinal fluid (AT1RB-aCSF).

Group 4: candesartan-streptozotocin (AT1RB-STZ).

### 2.3. Assessment of Learning and Memory

#### 2.3.1. Morris Water Maze Protocol

For the spatial memory assessment, on day 25, one group of rats was tested in the Morris water maze. This consisted of a circular water tank (150 cm in diameter and 60 cm high) filled with water to a height of 30 cm. The tank was divided into four imaginary quadrants (designated NW, NE, SW, SE), and in the one assigned as NW, a platform 10 cm in diameter was placed, submerged 2 cm below the water surface. Each of the room’s walls had a distinctive element, which the animals could use to locate the position of the escape platform and swim toward it. The rats were subjected to a training period, consisting of 4 sessions separated by 20 min (each starting from a different randomly selected starting point) per day for 3 days. Each session had a tolerance of 120 s to find the hidden platform. If the rat was unable to find the platform within that period of time, it was guided by the operator toward the platform and left there for 12 s. The time taken by each animal to find the platform was recorded as escape latency (in seconds). On day 4, twenty-four hours after the acquisition phase, the test was carried out, and the platform was absent. Rats were allowed to swim freely for 30 s, and the time spent in the target quadrant (where the platform was previously located) was recorded. This parameter was assessed in a blinded manner using Open Source Physics Tracker software (version 4.62) [16].

#### 2.3.2. Object Recognition Test Protocol

To analyse declarative memory, the object recognition test was assessed in another group of rats (on day 25). The apparatus consisted of a plastic box 60 × 60 × 30 cm (width, length, height), which had the floor covered with sawdust. The objects were made of plastic and existed in duplicate. They were never associated with a reward and were sufficiently heavy to prevent displacement by the animals. The procedure consisted of two habituation sessions separated by a 1 h interval, where the rats were allowed free exploration for 3 min. Twenty-four hours later, a habituation session was followed by the test session (3 min each), separated by a 1 h inter-session interval. During the test session, animals were presented with an identical copy of the familiar object (A) and a novel object (B). Exploration time was defined as the time spent touching an object with the snout. The discrimination index was defined as the exploration time of the novel object relative to the total exploration time of both objects [B/(A’ + B)]. This metric ranges from −1 to 1, where values near zero suggest an absence of discrimination between familiar and novel objects. Conversely, values approaching 1 reflect a robust preference for novelty, signifying that a representation of the familiar object has been successfully retained in the animal’s memory. To control for spatial bias, the location of the novel object was counterbalanced across animals. Animals showing a preference greater than 50% of the time for a specific location or object during the habituation phase were excluded. These behavioral metrics were recorded and quantified in a blinded manner through the systematic evaluation of video [11].

#### 2.3.3. Holeboard Test Protocol

Based on our previous work [13,17], on day 25, another group of animals was placed in a hole-board open field apparatus (60 cm × 60 cm × 30 cm). Behavior was video-recorded for 5 min, and head dips (defined as inserting the head into a hole) were manually scored in a blinded manner. A more homogeneous distribution of head dips reflects better spatial working memory performance, as the animal remembers previously explored holes in the short term and avoids re-exploring them. Conversely, a working memory deficit is indicated by a significant difference in exploration frequency between the two most explored holes. Consequently, statistical evaluation was restricted to the two primary holes visited by each subject. The exploration index was subsequently derived by determining the proportion of head dips into hole 1 (H1) relative to the combined total visits across both primary holes [H1/(H1 + H2)], with an identical calculation applied for hole 2.

### 2.4. Biochemical Analysis

#### 2.4.1. Brain Microvessel Isolation

On day 25, the animals are anesthetized and transcardially perfused with 250 mL of physiological saline and heparin. Upon completion of the perfusion, the brain was removed under cold conditions, cut into 1 mm^3^ pieces in 1 mL of cold HEPES-sucrose buffer (0.32 M sucrose; 3 mM HEPES; pH 7.4), and subsequently homogenized in 4 mL of cold HEPES-sucrose buffer. The resulting homogenate was centrifuged at 1000× *g* for 10 min at 4 °C, and the resulting pellet was resuspended in 2 mL of cold HEPES-sucrose buffer. It was centrifuged again at 1000× *g* for 10 min at 4 °C, discarding the upper half of the resulting supernatant. Then, 1 mL of cold HEPES-sucrose buffer was added, and the sample was centrifuged again at 100× *g* for 30 s at 4 °C; the supernatant was collected in a new tube and kept cold for later use. Separately, the resulting pellet was resuspended in 2.5 mL of cold HEPES-sucrose buffer and centrifuged again at 100× *g* for 30 s at 4 °C; the supernatant was collected and pooled with the previously stored supernatant. This last step was repeated once more. One milliliter of cold HEPES-sucrose buffer was added to the collected supernatants, and the mixture was centrifuged at 200× *g* for 2.5 min at 4 °C; the supernatant was discarded. Finally, the resulting pellet was resuspended in 3 mL of cold HEPES-sucrose buffer and centrifuged at 200× *g* for 2 min at 4 °C. A total of 2.5 mL of cold PBS-0.1% BSA was added to the resulting pellet, and the mixture was centrifuged at 200× *g* for 3 min at 4 °C; the supernatant was discarded, and 1 mL of cold PBS-BSA was added again. The sample was thoroughly homogenized, and an aliquot was set aside for the histomorphological evaluation of the isolated cerebral microvessels. The sample was then centrifuged at 14,000× *g* for 10 min at 4 °C, and the resulting pellet was stored at −70 °C until further use [18].

#### 2.4.2. Measurement of Malondialdehyde (MDA)

Ohkawa’s method [19] was used to determine the rate of lipid peroxidation in brain microvessels. Briefly, the pellet containing the microvessels was resuspended in 30 μL of KCl 1.15% and homogenized for 15 min on ice. A 15 μL aliquot was taken and mixed with SDS 8.1%, acetic acid 20%, pH 3.5, and TBA 0.8%. After 1 h of incubation at 95 °C, it was cooled under tap water for 5 min, then it was centrifuged for 10 min at 4000 rpm, and the absorbance of the pink coloured product obtained was read at 532 nm in a multiplate reader spectrophotometer. The MDA content was determined using a standard curve with tetrametoxypropane. Protein content of the samples was determined using the Bradford method [20].

#### 2.4.3. Collection of Cerebrospinal Fluid

The method described by Nirogi et al. [21] was followed. Briefly, rats were anesthetized, placed in a stereotaxic frame (Model 963 of 100 µm precision, David Kopf Instruments, Tujunga, CA, USA), and the position of the animal’s head was maintained downward at 45°. A 23-G needle connected to a draw syringe was inserted horizontally and centrally into the cisterna magna for CSF collection. A gentle aspiration made the CSF flow through the needle. Samples were put into 500 μL PE tubes and stored at −20 °C until use. A total of 4–6 animals per group were analysed [22]. This procedure was assessed on day 25.

#### 2.4.4. Cerebrospinal Fluid Analysis for Tumor Necrosis Factor-α (TNFα)

Rat TNFα was determined by ELISA using a commercial kit (BD OptEIA™ Rat TNF (Mono/Poly) ELISA Set) following the manufacturer’s protocol.

#### 2.4.5. Measurement of the Lateral Ventricles’ Volume

To measure ventricle volumes, after being anesthetized, the rats were decapitated, and the brain tissue was dissected out and kept in a 10% paraformaldehyde solution. After fixation, coronal sections 100 μm thick were cut with the help of a freezing microtome and picked up on slides. Between 10 and 15 sections, from bregma 2.2 to bregma −1.4, were stained with toluidine blue for contrast, and pictures were captured with a magnifying glass (30×) connected to a computer. Ventricular volumes were measured using the software IMAGE J (version 1.54) by taking measurements at different points of the bregma, and the total ventricular volume was determined by calculating the area under the curve.

### 2.5. Statistical Analysis

The study used two-way or three-way ANOVA with vehicle and AT1RB as pretreatment factors and aCSF and STZ as the second factor. For the water maze analysis, a two-way repeated-measures ANOVA was performed. If an interaction and/or main effect was observed, pairwise comparisons were made using the Bonferroni post-test. A value of *p* < 0.05 was considered significant. In the context of oxidative stress and histomorphological evaluations, where statistical significance was achieved, Cohen’s d test was used to calculate the magnitude of the effect size. Regarding TNF-α concentrations, Hedges’ g was applied to account for the limited number of samples analysed. Statistical processing of the data was conducted utilizing GraphPad Prism (version 8.0.2) and Jamovi (version 2.6.26) software packages. Outlier data were excluded following the Chauvenet criteria.

## 3. Results

### 3.1. Animal Weights

Neither oral administration of candesartan/vehicle nor ICV injection of STZ/aCSF affected the animals’ intake of food or water, as no significant difference was found in the animals’ weights during the treatment.

### 3.2. Holeboard Test

The statistical analysis indicates that the Veh-STZ group exhibited marked cognitive alterations. These were characterized by a significant disparity in exploration index between the two most explored holes, a hallmark of impaired short-term memory tracking and recall. Notably, the animals treated with candesartan did not show this cognitive impairment induced by the ICV STZ exposure. These results suggest that the AT1-R plays a central role in the working memory deficit induced by the ICV administration of STZ observed on day 25 (Figure 2A, Table 1). The AT1-R blockade alone did not affect working memory since the AT1RB-aCSF group did not show a statistically different exploration index between the primary holes. For more information regarding the statistical significance, refer to Supplementary Table S1.

### 3.3. Novel Object Recognition Test

The statistical analysis showed that the Veh-STZ group exhibited a reduced discrimination index for novel object recognition compared to its control group. The pharmacological intervention with candesartan successfully prevented this cognitive impairment, as the performance of the AT1RB-STZ group remained indistinguishable from control levels, but was significantly elevated compared to the Veh-STZ group (Figure 2B, Table 1). Our results suggest that the AT1-R plays a key role in the declarative memory deficit induced by the ICV administration of STZ, evidenced on day 25. The candesartan administration alone did not affect declarative memory, as the discrimination index of the AT1RB-aCSF group was not statistically different from the Veh-aCSF group. Also, the evaluation of total exploration time revealed no significant variations across the experimental groups. For further details concerning the statistical relevance of this behavioral analysis, please consult Supplementary Table S1.

### 3.4. Morris Water Maze Test

In the specific time frame employed, ICV-administered STZ did not affect spatial navigation or long-term memory performance when examined through the Morris water maze task on day 25. In this sense, no significant differences were observed during the twelve training sessions (Supplementary Table S1) nor in the test trial performed 24 h after the last training session (Table 1). For more information regarding the statistical significance, refer to Supplementary Table S1.

### 3.5. MDA Level in Brain Microvessels

The statistical analysis showed that ICV-administered STZ induced a significant increase in MDA levels in brain microvessels on day 25, compared with control groups. Pharmacological intervention with candesartan blunted this alteration induced by ICV STZ administration (Figure 2C, Table 2). Furthermore, AT1RB alone did not affect MDA levels, as the values of the AT1RB-aCSF group were not statistically different from the Veh-aCSF group. For further details concerning the statistical relevance of this biochemical analysis, please consult Supplementary Table S2.

### 3.6. TNFα in Cerebrospinal Fluid

Central STZ administration triggered a significant elevation in CSF TNF-α levels on day 25, compared with control groups. The animals treated with candesartan did not show this inflammatory surge induced by ICV STZ, suggesting a key role for the AT1-R (Figure 2D, Table 2). AT1RB alone did not affect TNF-α levels, as the values of the AT1RB-aCSF group were not statistically different from the Veh-aCSF group. Nevertheless, future investigations involving more extensive cohorts remain imperative to further substantiate the findings concerning TNF-α concentrations. Further statistical details are provided in Supplementary Table S2.

### 3.7. Histomorphological Findings

With respect to the volume of the lateral ventricles, statistical analysis showed significant differences only between the Veh-STZ and AT1RB-STZ groups (Figure 2E, Table 2). Although this STZ exposure elicited marked differences between vehicle- or candesartan-treated groups, no statistically significant variations emerged when compared to their respective control groups. For further details concerning the statistical relevance of this histomorphological analysis, please consult Supplementary Table S2.

## 4. Discussion

Our results suggest that AT1-R is involved in the declarative and working memory deficit induced by ICV-administered STZ, embedded in the time frame chosen for an animal model of sAD. To model and assess diverse cognitive domains, we employed the object recognition test for declarative memory and the holeboard task for working memory assessment; furthermore, the Morris water maze was used to examine spatial and long-term memory performance. Regarding the object recognition test, where the preference for the new object suggests that a representation of the old object has been formed in the animal’s memory [10,11,23,24], the ICV-administered STZ induced a declarative memory deficit, which was prevented by the AT1-R blockade. Previously, our laboratory showed the involvement of AT1-R in declarative memory processes since candesartan prevented the alterations observed when the same cognitive paradigm was used in schizophrenia models, induced by ketamine and amphetamine administration [10,11]. Similar results pointing out the AT1-R role were observed in the holeboard test, where working memory scores measure a rat’s ability to recall and track short-term information within a single trial [13,17]. In the present work, coincidentally, the results show that AT1-R is involved in the impairment of working memory triggered by the ICV administration of STZ. Finally, spatial navigation is a task that involves integrating not only spatial cues, but also cues from the animal’s own body, proprioception, and the effort exerted. In the timeframe used in the present protocol, ICV-administered STZ did not affect spatial and long-term memory. Although preserved performance in the Morris water maze task does not independently characterize early-stage sAD, the concurrent impairments in working and declarative memory, together with initial surges in lipid peroxidation and TNF-α concentrations (discussed below), suggest that this experimental paradigm models the incipient phases of sAD progression. Accordingly, the work of Baerends et al. [25] indicates that early stages of sAD are characterized by preserved spatial navigation capabilities, coinciding with minor hippocampal and lateral entorhinal cortex alterations. Limited investigations have focused on the initial stages of sAD; nevertheless, characterizing the incipient alterations in the progression of this brain pathology remains imperative to broaden the therapeutic window and achieve long-term functional improvements.

The extensive literature has characterized the neuroinflammatory cascades within experimental paradigms of sAD and vascular dementia, correlating these immune responses with the progression of underlying neuropathological hallmarks [1]. Furthermore, it has been described that brains from AD patients have elevated levels of inflammatory proteins such as TNFα that may contribute to the degenerative process [26]. Consistent with these findings, a significant elevation in both TNF-α and MDA concentrations was observed within the specific temporal window of this experimental sAD paradigm. It is imperative to consider that cerebrovascular dysregulation is recognized as a primary inciting factor for numerous neuroinflammatory states, given its capacity to compromise regional metabolic homeostasis and subsequent neuronal integrity [27]. However, at the time point evaluated, we did not find differences in GFAP expression in the hippocampus or caudate putamen. Nevertheless, future investigations involving more extensive cohorts remain imperative to further substantiate the findings concerning TNF-α concentrations. Regarding histomorphological analysis, the STZ-induced alteration in ventricular volume did not reach statistical significance compared with control groups; however, the ventricular volume in the animals treated with candesartan and STZ was different compared with the animals who receive only STZ. The lack of statistical significance relative to control cohorts might conceivably stem from the specific temporal window or the magnitude of the observed variation. To date, there is no prior evidence describing this effect in relation to STZ administration. The ventricular enlargement could be a consequence of inflammation leading to vasogenic edema, a phenomenon commonly observed in an inflammatory central nervous system environment. Additionally, these alterations might stem from STZ-induced neurotoxicity and the associated cellular loss observed across the hippocampus, cerebral cortex, and periventricular regions [7,8,12,25]. Remarkably, the observed divergence following STZ administration between the vehicle- and candesartan-treated cohorts represents an intriguing phenomenon that warrants further investigation. The observed increase in TNF-α and MDA levels could be related to these described STZ-induced processes. Crucially, our findings suggest that central AT1-R plays a key role in microvascular oxidative stress and TNFα concentrations within this experimental paradigm induced by STZ. In the cerebral microvasculature, Ang II drives endothelial dysfunction and increases reactive oxygen species specifically through the direct upregulation of the Nox2 subunit of NADPH oxidase. Consequently, this vascular impairment and amplified inflammatory response represent the combined outcome of AT1-R activation, inflammatory mediators, and ROS [28,29,30]. Coincidentally, we previously showed the key role of AT1-R in oxidative stress responses and neuroinflammatory processes in different brain structures with microvessel compromise induced by psychostimulant administration. In the same way, the well-described exacerbation of brain microvascular proinflammatory markers in spontaneously hypertensive rats is abolished by AT1-R antagonism [31]. Recent findings reported that candesartan effectively reduced IL-1β, IL-6, and TNF-α concentrations, alongside microglial activation, in a murine model of perioperative neurocognitive disorder linked to hypertension and neuroinflammatory cascades [32]. In peripheral systems, pharmacological inhibition of AT1-R prevents stress-triggered gastric ulcerations through the preservation of regional blood perfusion and the attenuation of sympathoadrenal cascades. This gastroprotection was fundamentally linked to the downregulation of TNF-α and ICAM-1 signaling, which subsequently prevents neutrophil infiltration while preserving local PGE2 secretion and homeostatic glucocorticoid responses [33]. On the other hand, broad evidence supports the neuroprotective effect of candesartan given its role as a peroxisome proliferator-activated receptor-gamma (PPARγ) agonist [34]. Furthermore, it is important to consider that AT1-R inhibition leads to increased circulating levels of Ang II, which are then available for binding to AngII type 2 and 4 receptors. Activation of these receptors has been shown to reduce oxidative stress and neuroinflammation, as well as to improve cerebral blood flow in animal models [9].

In addition to sAD, increased life expectancy and elevated stress levels in modern society are associated with a higher risk of hypertension as well as learning and memory impairments. AT1-R blockers are widely prescribed antihypertensive agents with proven efficacy in managing hypertension and cardiovascular diseases [35]. Moreover, as pointed out in a recent review by Saavedra [36], in his words, “AT1 tor blockers are not just for hypertension anymore”. In this sense, our results contribute to a growing body of evidence supporting the central Ang II system as a promising therapeutic target and reinforce the potential for repurposing AT1-R antagonists in the prevention and treatment of neurological disorders to improve patient quality of life. Given the population’s increased life expectancy, it is important to select drugs that, in addition to their antihypertensive action, offer protection against the harmful effects associated with aging and are also suitable for use in normotensive individuals.

## 5. Conclusions and Limitations

Our findings show that AT1-R is involved in mediating neurotoxic cascades and cognitive decline within the specific timeframe of this experimental sAD model. The study shows evidence of alterations in TNF-α in cerebrospinal fluid and malondialdehyde expression in isolated microvessels, while also suggesting alterations in ventricular volume associated with STZ administration. At the time point selected for the Morris water maze test, we found no differences. Therefore, further studies are required to determine whether the result obtained is a consequence of the timing chosen for the evaluation. Additionally, our study design investigated the involvement of AT1-R in central STZ-triggered neuroinflammatory cascades and cognitive deficits, targeting preventive strategies. Subsequent studies are required to establish if candesartan administration truly arrests disease progression or simply postpones behavioral manifestations. Also, further research remains crucial to clarify whether AT1-R blockade can effectively reverse pre-existing neurodegenerative deficits after the clinical onset. In a broader context, concerning the brain renin-angiotensin pathway via AT1-R, thorough research remains imperative to fully characterize the underlying mechanisms of neuroinflammation and related memory deficits.

Furthermore, as this investigation exclusively focused on male subjects, and considering that sexual dimorphism significantly influences both sAD pathogenesis and renin-angiotensin system regulation, additional studies are imperative to elucidate these gender-specific variations.

## Figures and Tables

**Figure 1:**
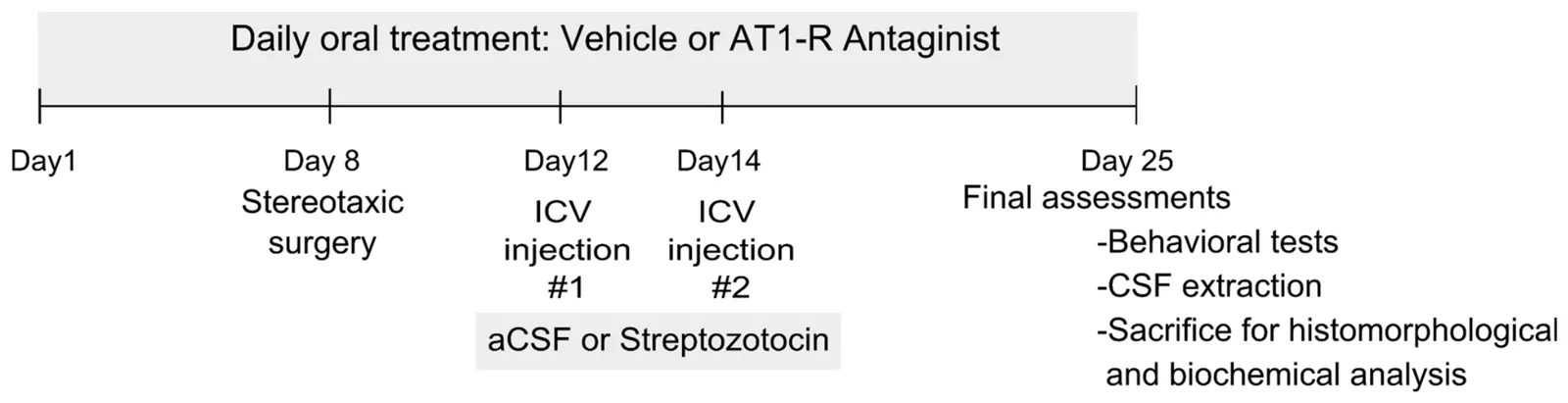
Experimental protocol used in the present work. # First and second administration of STZ/aCSF.

**Figure 2:**
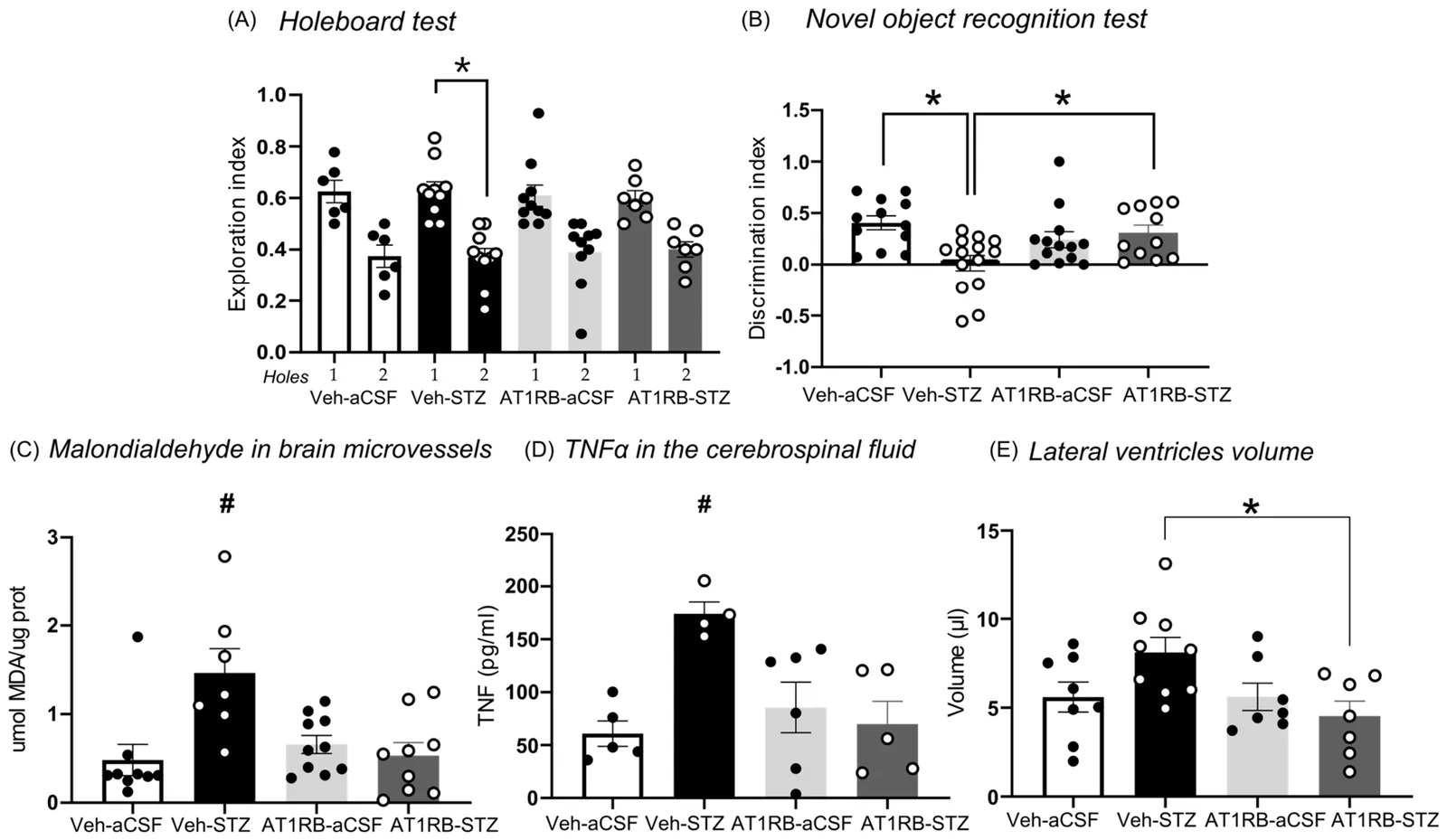
Role of AT1-R in biochemical and histomorphological alterations induced by ICV-administered STZ observed on day 25. (**A**) Working memory was evaluated by the exploration index in the holeboard test. The X-axis represents the two primary holes. (**B**) Declarative memory was evaluated by the discrimination index for the novel object. (**C**) Malondialdehyde level in brain microvessels. Data are expressed as μmol MDA/μg prot. (**D**) TNFα level in the cerebrospinal fluid, expressed in pg/mL. (**E**) Lateral ventricles volume, expressed in μL. Data are shown as Mean ± SEM. * *p* < 0.05. # Different from all other experimental groups (*p* < 0.05). The black and white dots serve only to enhance image visualization.

**Table 1:** Summary of descriptive data and statistical outcomes from behavioral assessments performed on experimental day 25. Only statistically significant differences (*p* < 0.05) are included. For more details, see Supplementary Table S1.

Holeboard Test				
Experimental groups	n	Exploration index (%)		Bonferroni’s post hoc Hole 1 vs. Hole 2 Adjusted *p* value
		Hole 1: Mean ± SEM	Hole 2: Mean ± SEM	
Veh-aCSF	6	62.5 ± 4.3	37.5 ± 4.3	
Veh-STZ	10	62.9 ± 3.4	37.0 ± 3.4	*p* = 0.024
AT1RB-aCSF	10	60.9 ± 4.2	39.1 ± 4.2	
AT1RB-STZ	7	59.8 ± 2.9	40.1 ± 2.9	
Three animals were excluded from the Veh-aCSF group, and two from the AT1RB-STZ group, by the Chauvenet criteria.				
Novel object recognition test				
Experimental groups	n	Discrimination index Mean ± SEM	Bonferroni’s post hoc	Adjusted *p* value
Veh-aCSF	12	0.41 ± 0.07	Veh-STZ vs. Veh-aCSF	*p* = 0.002
Veh-STZ	13	0.014 ± 0.07		
AT1RB-aCSF	12	0.24 ± 0.08	Veh-STZ vs. AT1RB-STZ	*p* = 0.036
AT1RB-STZ	12	0.31 ± 0.07		
Three animals were excluded from the Veh-aCSF group, and one from Veh-STZ, following the Chauvenet criteria.				
Water Morris maze test				
Experimental groups	n	Exploration time (sec) Mean ± SEM		
Veh-aCSF	15	10.8 ± 0.96		
Veh-STZ	14	10.73 ± 1.23		
AT1RB-aCSF	13	9.54 ± 1.14		
AT1RB-STZ	11	11.67 ± 1.08		
No animals were excluded from the analysis.				

**Table 2:** Summary of statistical findings derived from biochemical and histomorphological evaluations performed on experimental day 25. Only statistically significant differences (*p* < 0.05) are included. For more details, see Supplementary Table S2.

MDA Levels in Brain Microvessels					
Experimental groups	n	Mean ± SEM	Bonferroni’s post hoc	Adjusted *p*-value	Cohen’s D test
Veh-aCSF	10	0.48 ± 0.17	Veh-STZ vs. Veh-aCSF	*p* = 0.001	d = 1.57
Veh-STZ	7	1.46 ± 0.27	Veh-STZ vs. AT1RB-aCSF	*p* = 0.017	d = 1.53
AT1RB-aCSF	10	0.65 ± 0.10			
AT1RB-STZ	9	0.53 ± 0.15	Veh-STZ vs. AT1RB-STZ	*p* = 0.005	d = 1.59
No animal was excluded from this experiment.					
TNFα in Cerebrospinal Fluid					
Experimental groups	n	Mean ± SEM	Bonferroni’s post hoc	Adjusted *p*-value	Hedges’ g; [IC 95%]
Veh-aCSF	5	60.9 ± 11.9	Veh-STZ vs. Veh-aCSF	*p* = 0.009	3.95; [1.42, 6.48]
Veh-STZ	4	174.0 ± 11.2	Veh-STZ vs. AT1RB-aCSF	*p* = 0.04	1.65; [0.34–2.97]
AT1RB-aCSF	6	85.6 ± 23.9			
AT1RB-STZ	5	69.0 ± 21.5	Veh-STZ vs. AT1RB-STZ	*p* = 0.016	2.32; [0.79–3.84]
One animal was excluded from the Veh-STZ group and one from the AT1RB-STZ group following the Chauvenet criteria.					
Volume of lateral ventricles					
Experimental groups	n	Mean ± SEM	Bonferroni’s post hoc	Adjusted *p*-value	Cohen’s D test
Veh-aCSF	8	5.6 ± 0.8	Veh-STZ vs. AT1RB-STZ	*p* = 0.031	d = 1.47
Veh-STZ	9	8.1 ± 0.8			
AT1RB-aCSF	7	5.6 ± 0.8			
AT1RB-STZ	7	4.5 ± 0.8			
No animal was excluded from this experiment.					

## Data Availability

The original data presented in the study are openly available in Repositorio Digital de la Universidad Nacional de Córdoba at https://rdu.unc.edu.ar 24 September 2026.

## Supplementary Materials

The following supporting information can be downloaded at: https://www.mdpi.com/article/10.3390/cimb48100998/s1.

## Abbreviations

The following abbreviations are used in this manuscript:

sAD	Sporadic Alzheimer’s disease
STZ	Streptozotocin
ICV	Intracerebroventricular
AngII	Angiotensin II
AT1-R	Angiotensin II type 1 receptors
AT1RB	Angiotensin II type 1 receptor blocker.
Veh	Vehicle
aCSF	Artificial cerebrospinal fluid
MDA	Malondialdehyde
