# Supplementary material for: Brain AT1 Receptor Involvement in Cognitive Decline and Neuroinflammation in Streptozotocin-Treated Rats
Source: Curr Issues Mol Biol. 2026 Sep 28;48(10):998. doi: 10.3390/cimb48100998 (PMC13703344; doi:10.3390/cimb48100998)
Supplement: Supplementary file 1 [file cimb-48-00998-s001.zip › cimb-4517501-Supplementary.pdf]

Supplementary Table S1: Statistical results obtained from behavioral evaluations.

Holeboard test

Three-way ANOVA		
Source of Variation	F (DFn, DFd)	P value
Holes	F (4, 116) = 46.91	P<0.0001
Treatment	F (1, 29) = 0.333	P=0.568
Drug	F (1, 29) = 0.145	P=0.706
Holes x Treatment	F (4, 116) = 0.557	P=0.694
Holes x Drug	F (4, 116) = 2.842	P=0.027
Treatment x Drug	F (1, 29) = 2.034	P=0.164
Holes x Treatment x Drug	F (4, 116) = 1.227	P=0.303
Bonferroni's post hoc		Adjusted P value
Veh-STZ: hole 1 vs. hole 2		p<0.0001
Veh-aCSF: hole 1 vs. hole 2		p>0.05
AT1RB-aCSF: hole 1 vs. hole 2		p>0.05
AT1RB-STZ: hole 1 vs. hole 2		p>0.05

Notes: Treatment: consider Vehicle vs Candesartan

Drug: Saline vs Streptozotocin

Holes: the five holes in the holeboard

Bonferroni's post hoc test compared each cell mean with every other cell mean. For clarity, this table shows only the comparisons between the first and second most explored holes for each experimental group.

Novel object recognition test

Discrimination index

Two-way ANOVA		
Source of Variation	F (DFn, DFd)	P value
Interaction	F (1, 46) = 9.569	P=0.003
Treatment	F (1, 46) = 0.787	P=0.379
Drug	F (1, 46) = 4.746	P=0.034
Bonferroni's post hoc		Adjusted P value
Veh-aCSF vs. Veh-STZ		p=0.002
Veh-aCSF vs. AT1RB-aCSF		p=0.408
Veh-aCSF vs. AT1RB-STZ		p=0.816
Veh-STZ vs. AT1RB-aCSF		p=0.123
Veh-STZ vs. AT1RB-STZ		p=0.036
AT1RB-aCSF vs. AT1RB-STZ		p=0.920

Total exploration time

Two-way ANOVA		
Source of Variation	F (DFn, DFd)	P value
Interaction	F (1, 46) = 0.006	P=0.937
Treatment	F (1, 46) = 0.6	P=0.442
Drug	F (1, 46) = 0.011	P=0.916

Notes: Treatment: consider Vehicle vs Candesartan

Drug: Saline vs Streptozotocin

Water Morris maze test

Training trials

Three-way ANOVA

Test trial

Two-way ANOVA

Source of Variation	F (DFn, DFd)	P value
Trials	F (11, 550) = 60.30	P<0.0001
Treatment	F (1, 50) = 0.73	P=0.397
Drug	F (1, 50) = 2.194	P=0.145
Trials x Treatment	F (11, 550) = 0.979	P=0.464
Trials x Drug	F (11, 550) = 1.790	P=0.053
Treatment x Drug	F (1, 50) = 0.1659	P=0.685
Trials x Treatment x Drug	F (11, 550) = 0.485	P=0.913

Source of Variation	F (DFn, DFd)	P value
Interaction	F (1, 50) = 0.623	P=0.434
Treatment	F (1, 50) = 0.106	P=0.746
Drug	F (1, 50) = 1.193	P=0.280

Notes: Treatment: consider Vehicle vs Candesartan

Drug: Saline vs Streptozotocin

Trials: the 12 trials performed in the training session of the water Morris maze.

Supplementary Table S2: Statistical results obtained from biochemical and histopathological evaluations.

Malondialdehyde level in brain microvessels

Two-way ANOVA		
Source of Variation	F (DFn, DFd)	P value
Interaction	F (1, 32) = 11.49	P=0.002
Treatment	F (1, 32) = 4.390	P=0.044
Drug	F (1, 32) = 6.995	P=0.013
Bonferroni's post hoc		Adjusted P value
Veh-aCSF vs. Veh-STZ		0.001
Veh-aCSF vs. AT1RB-aCSF		>0.999
Veh-aCSF vs. AT1RB-STZ		>0.999
Veh-STZ vs. AT1RB-aCSF		0.017
Veh-STZ vs. AT1RB-STZ		0.005
AT1RB-aCSF vs. AT1RB-STZ		>0.999

TNFα in Cerebrospinal Fluid

Two-way ANOVA		
Source of Variation	F (DFn, DFd)	P value
Interaction	F (1, 16) = 10.55	P=0.0050
Treatment	F (1, 16) = 4.008	P=0.0625
Drug	F (1, 16) = 6.028	P=0.0259
Bonferroni's post hoc		Adjusted P value
Veh-aCSF vs. Veh-STZ		0.009
Veh-aCSF vs. AT1RB-aCSF		>0.999
Veh-aCSF vs. AT1RB-STZ		>0.999
Veh-STZ vs. AT1RB-aCSF		0.039
Veh-STZ vs. AT1RB-STZ		0.016
AT1RB-aCSF vs. AT1RB-STZ		>0.999

Volume of lateral ventricles

Two-way ANOVA		
Source of Variation	F (DFn, DFd)	P value
Interaction	F (1, 27) = 4.529	P=0.0426
Treatment	F (1, 27) = 4.459	P=0.0441
Drug	F (1, 27) = 0.71	P=0.4068
Bonferroni's post hoc		Adjusted P value
Veh-aCSF vs. Veh-STZ		0.215
Veh-aCSF vs. AT1RB-aCSF		>0.999
Veh-aCSF vs. AT1RB-STZ		>0.999
Veh-STZ vs. AT1RB-aCSF		0.261
Veh-STZ vs. AT1RB-STZ		0.031
AT1RB-aCSF vs. AT1RB-STZ		>0.999
